# Genome-wide diversity and demographic dynamics of Cameroon goats and their divergence from east African, north African, and Asian conspecifics

**DOI:** 10.1371/journal.pone.0214843

**Published:** 2019-04-19

**Authors:** Getinet Mekuriaw Tarekegn, Patrick Wouobeng, Kouam Simo Jaures, Raphael Mrode, Zewdu Edea, Bin Liu, Wenguang Zhang, Okeyo Ally Mwai, Tadelle Dessie, Kassahun Tesfaye, Erling Strandberg, Britt Berglund, Collins Mutai, Sarah Osama, Asaminew Tassew Wolde, Josephine Birungi, Appolinaire Djikeng, Félix Meutchieye

**Affiliations:** 1 Department of Animal Breeding and Genetics, Swedish University of Agricultural Sciences, Uppsala, Sweden; 2 Department of Animal Production and Technology, Bahir Dar University, Bahir Dar, Ethiopia; 3 Biosciences Eastern and Central Africa-International Livestock Research Institute (BecA-ILRI) Hub, Nairobi, Kenya; 4 Faculty of Agronomy and Agriculture, University of Dschang, Dschang, Cameroon; 5 International Livestock Research Institute (ILRI), Nairobi, Kenya; 6 Department of Animal Science, Chungbuk National University, Cheongju, Korea; 7 Nei Mongol BioNew Technology Co.Ltd, Hohhot, China; 8 College of Animal Science, Inner Mongolia Agricultural University, Hohhot, China; 9 International Livestock Research Institute (ILRI), Addis Ababa, Ethiopia; 10 Department of Microbial, Cellular and Molecular Biology, Addis Ababa University, Addis Ababa, Ethiopia; 11 The University of Queensland, Queensland, Australia; 12 Centre for Tropical Livestock Genetics and Health, The University of Edinburgh, Scotland, United Kingdom; National Cheng Kung University, TAIWAN

## Abstract

Indigenous goats make significant contributions to Cameroon’s national and local economy, but little effort has been devoted to identifying the populations. Here, we assessed the genetic diversity and demographic dynamics of Cameroon goat populations using mitochondrial DNA (two populations) and autosomal markers (four populations) generated with the Caprine 50K SNP chip. To infer genetic relationships at continental and global level, genotype data on six goat populations from Ethiopia and one population each from Egypt, Morocco, Iran, and China were included in the analysis. The mtDNA analysis revealed 83 haplotypes, all belonging to haplogroup A, in Cameroon goats. Four haplotypes were shared between goats found in Cameroon, Mozambique, Namibia, Zimbabwe, Kenya, and Ethiopia. Analysis of autosomal SNPs in Cameroon goats revealed the lowest *H*_O_ (0.335±0.13) and *H*_E_ (0.352±0.15) in the North-west Highland and Central Highland populations, respectively. Overall, the highest *H*_O_ (0.401±0.12) and *H*_E_ (0.422±0.12) were found for Barki and Iranian goats, respectively. Barki goats had the highest average MAF, while Central Highland Cameroon goats had the lowest. Overall, Cameroon goats demonstrated high *F*_IS_. AMOVA revealed that 13.29% of the variation was explained by genetic differences between the six population groups. Low average *F*_ST_ (0.01) suggests intermixing among Cameroon goats. All measures indicated that Cameroon goats are closer to Moroccan goats than to other goat populations. PCA and STRUCTURE analyses poorly differentiated the Cameroon goats, as did genetic distance, Neighbor-Net network, and neighbor-joining tree analyses. The haplotype analysis of mtDNA showed the initial dispersion of goats to Cameroon and central Africa from north-east Africa following the Nile Delta. Whereas, the approximate Bayesian computation indicated Cameroon goats were separated from Moroccan goats after 506 generations in later times (~1518 YA), as supported by the phylogenetic net-work and admixture outputs. Overall, indigenous goats in Cameroon show weak phylogenetic structure, suggesting either extensive intermixing.

## Introduction

Goats are an important economic resource in Africa. For instance, in Cameroon there are 6.2 million goats [1]. They, together with sheep, provide 21% of the meat demand [2] for 22.8 million people [3]. Their small size, low maintenance requirements, short generation interval, good prolificacy, hardiness, and potential to adapt a wide range of agro-ecological zones have made goats the most important livestock species for many smallholder farmers and pastoral communities [4, 5]. African goats are also known for their heat tolerance (e.g., Egyptian Barki, Sahelian, and Sudanese goat populations) and trypanotolerance (e.g., West African Dwarf) [6–8]. A few goat populations, such as the Nigerian Dwarf goat, a variant of the West African Dwarf, are well-known for their prolificacy. Goats are also important sources of milk and meat and provide financial stability to pastoral communities and smallholder farmers in arid, semi-arid, and mountainous areas of Africa [9–13].

Goats (*Capra aegagrus*) were among the first species to be domesticated, about 10,500 years ago [14–16], and dispersed to various parts of the world [17]. Archaeological data indicate that goats were first introduced to Africa via the Mediterranean Sea coast, Red Sea Hills, and overland via the Sinai Peninsula and Nile Delta around 7000 years ago [18, 19]. The shared haplotypes observed for Ethiopian, Egyptian, and Saudi Arabian indigenous goat populations in a recent mitochondrial DNA (mtDNA) study confirm these routes of dispersion [20].

Modern maternal DNA analysis shows that, of six globally identified mtDNA haplogroups, three (A, B and G) are present in Africa [21, 22]. Haplogroup A has been observed in all regions of the continent, haplogroup B is limited to South Africa and Namibia [22], and haplogroup G has been reported in Egypt [22], Kenya [23], Somalia and Sudan [24, 25], and Ethiopia [20]. However, there is no documented information on the maternal origin of the goat populations in many African countries. Similarly, only a few autosomal marker-based studies have been conducted to characterize the indigenous goats in Africa. In Ethiopia and selected sub-Saharan African countries, studies on the genetic diversity of indigenous goats using microsatellite (SSR) markers have revealed low levels of differentiation among the goat populations [26, 27]. Similarly, studies by Guangul [28] and Tarekegn [29] on the genetic diversity and population structure of two and 14 Ethiopian goat populations, respectively, using 50K SNP chip panel, have both revealed a high level of admixture. High admixture status has also been reported for indigenous goat populations in Morocco and Sudan [30, 31].

In a previous study in Cameroon, eight indigenous goat populations were characterized using SSR markers and, based on the results, these populations were regrouped to four [32], despite the fact that principal component analysis (PCA) in the same study did not show a clear classification pattern. This could be related to lower abundance, in the genome, of the SSR markers used to uncover the structure of the study populations [33]. This suggests that highly abundant, high-resolution markers should be used to unravel the genetic diversity and population structure of indigenous goats in Cameroon, which in turn could help in designing appropriate breeding strategies. Therefore, the aim of this study was to describe the genetic diversity and demographic dynamics of Cameroon goat populations using mitochondrial DNA and genome-wide autosomal markers.

## Materials and methods

Sampling for this work was conducted in close collaboration with the Directorate of Veterinary Services under the supervision of the Ministry of Livestock in Cameroon. No animal sacrifice was required; hair samples were collected from 388 animals owned by smallholder farmers in rural areas of Cameroon (S1 Table).

### Sampling, DNA extraction, PCR amplification and sequencing

Genomic DNA was extracted from four indigenous goat populations in Cameroon (Central Highland, North-west Highland, Forest, Djallonke) using the Qiagen DNA extraction protocol and DNeasy®Blood & Tissue Kit (http://diagnostics1.com/MANUAL/General_Qiagen.pdf). Samples from the Djallonke (n = 48) and North-west Highland (n = 45) goat populations were used for mtDNA analysis. The PCR amplification steps, PCR reaction concentrations, and PCR programs, including the sequencing technology, were carried out as described in Tarekegn et al. [20]. For analysis of autosomal marker-based variation, 324 animals representing all four populations of Cameroon indigenous goats (Central Highland: n = 94; North-west Highland: n = 166; Forest goat: n = 31, and Djallonke: n = 33) were genotyped using the Caprine 50K SNP chip panel [34]. For comparison, we included genotype data from six Ethiopian indigenous goat populations (Ambo: n = 119; Afar: n = 49; Keffa: n = 51; Gumez: n = 42; Long-eared Somali: n = 48; Nubian: n = 47), representing East Africa; Egyptian Barki goat (n = 52) and Moroccan goat (n = 30), representing north Africa; one population from Iran (n = 9), and Cashmere goat (n = 108) from China. In total, 848 animals were included in the analysis. The inclusion of Ethiopian goats took into account two main agro-climates. The Gumez and Keffa populations are found in the humid environment of north-west and southern Ethiopia, while the Nubian, Afar, and Long-eared Somali goats derive from the dry and arid environments of north, north-east, and south-east regions of Ethiopia. The Keffa and Gumez populations were also used to test the hypothesis that the genotypes of goat populations found in the north, west, and south of Ethiopia are influenced by West African Dwarf and Central African Dwarf goat genotypes [35]. Furthermore, the north and east regions of Ethiopia were assumed to be the initial entry points of goats into the country [20, 29].

### Data analysis

#### The *d-loop* sequence

All chromatograms were visualized using CLC Workbench 7.0.4 (CLC Bio-Qiagen). The sequence fragments were edited manually using MEGA6 [36] to correct possible base calling errors. Multiple sequence alignments were performed in CLC Workbench with the ClustalW algorithm [37] and variable sites were scored against the *C*. *hircus* reference sequence (GenBank accession number: GU223571: direct submission). In total, we generated 93 sequences and determined the number of haplotypes with DnaSP v5 10.01 [38]. The level of genetic diversity represented by the number of haplotypes, haplotype diversity (*H*d), nucleotide diversity (π), and mean number of nucleotide differences between haplotypes and their standard deviations were determined for each goat population and across all populations, using ARLEQUINv3.5.2 [39].

To visualize the genetic relationship between individuals and populations, a phylogenetic tree was constructed using all the haplotypes with the Neighbor-Joining (NJ) algorithm implemented in MEGA6. The level of confidence associated with each bifurcation was evaluated with 1000 bootstrap replications. To complement the NJ tree, obtain further insights into the genetic relationships between the haplotypes, and determine the number of distinct mtDNA *d*-loop haplogroups present in the dataset, the median-joining (MJ) network [40] was constructed using Network v4.6 (www.fluxus-engineering.com). All mutations and character states were equally weighted. To visualize the variation and the gene flow in Cameroon goats in the context of the global caprine variation, 570 sequences from 29 countries representing the six globally defined mtDNA *d*-loop haplogroups [22] were retrieved from GenBank (S2 Table). The reference sequences for each haplogroup were incorporated into the NJ phylogenetic and MJ network analysis. These analyses were limited to the 481 bp of the first hypervariable (HVI) region of the mtDNA *d*-loop [21], which corresponded to positions 15709–16190 bp of the *C*. *hircus* mitochondrial reference sequence (GenBank accession number: GU295658).

Population demographic history and dynamics were inferred from haplotype mismatch distribution patterns [41] for each population and the haplogroups revealed by the MJ network analysis. Departures of the observed sum of squares differences (*SSD*) from the simulated model of demographic or spatial expansion were tested with the Chi-square test of goodness of fit statistic and Harpending’s raggedness index “*r*” [42], following 1000 coalescent simulations. Analysis of mismatched distribution patterns was performed with two coalescent-based estimators of neutrality, Fu’s *F*_S_ [43] and Tajima’s *D* [44]. The significance of these two statistics was tested with 1000 coalescent simulations implemented in ARLEQUIN v3.5.2.

#### Single nucleotide polymorphism (SNP) genotypes

All the four Cameroon goat populations analyzed and the 10 reference populations were genotyped using the caprine 50K SNP BeadChip array (Illumina Inc., San Diego, CA) [34]. Autosomal SNPs were filtered for call rate by sample and marker ≥ 90% and minor allele frequency (MAF) ≥ 5%. After applying these quality control criteria, 43421 SNPs for 848 animals remained for analysis. To avoid the effects of ascertainment bias on the level of admixture, these 43421 SNPs were subjected to linkage disequilibrium (LD) pruning, leaving 7930 SNPs for population structure analysis. However, based on the above filtering criteria Forest goat population showed that 95.41% of the markers for this population were monomorphic loci, and therefore we excluded this population from further downstream analysis.

### Statistical analysis

#### Genetic diversity and differentiation

Within-breed genetic diversity, represented as observed heterozygosity (*H*_O_), expected heterozygosity (*H*_E_), inbreeding coefficient (*F*_*IS*_), and deviation from Hardy-Weinberg equilibrium (HWE), was determined with ARLEQUIN v3.5.2. Analysis of molecular variance (AMOVA) was performed using the same software to apportion genetic variance within and between populations and among groups. The goat populations were considered as a group based on their country of origin. The distribution of MAF was assessed based on the following categories: fixed alleles (MAF = 0.00), rare alleles (0 < MAF < 0.05), intermediate alleles (0.05 ≤ MAF < 0.10), common alleles (0.10 ≤ MAF ≤ 0.5), and polymorphic SNPs (MAF > 0.01).

#### Genetic relationships and population structure

Population pairwise genetic differentiations **(***F*_ST_) [45] and Reynolds’ genetic distances [46] were estimated with ARLEQUIN v3.5.2. A Neighbor-Net network and NJ phylogenetic tree were constructed using the pairwise genetic distances with SplitsTree ver.4.10 [47] and TASSEL [48], respectively. To further investigate genetic relationships, we carried out PCA using allele frequency data with the SNPRelate R package [49, 50]. The population genetic structure based on SNP information was evaluated using STRUCTURE v.2.3.4 [51] assuming hypothetical population clusters (*K*) ranging between 2 and 15. For each *K*-value, we carried out five runs of 20,000 Markov chain Monte Carlo iterations after a burn-in of 10,000 iterations. The STRUCTURE output was further analyzed in STRUCTURE HARVESTER [52] and the Δ*K* method [53] was used to determine the optimal number of genetic groups present in the dataset.

#### Approximate Bayesian Computation (ABC) simulations

**Fig 1:**
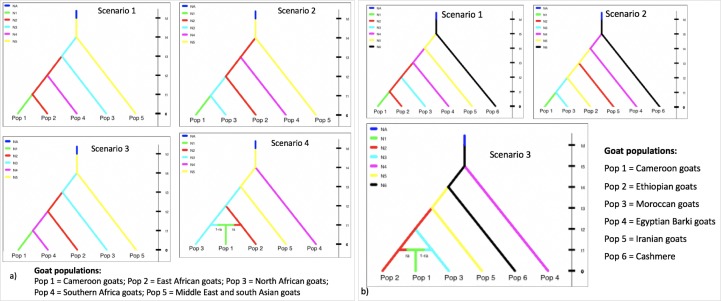
Regional representation of goat populations to infer the historical gene flow towards Cameroon examined using Approximate Bayesian Computation implemented in DIYABC. a) Based on mtDNA, Cameroon goats were descended either from east Africa (scenario 1), north Africa (scenario 2), southern Africa (scenario 3) or east and north Africa (scenario 4). b) Based on autosomal SNP markers, Cameroon goats were descended either from east Africa (Ethiopia; scenario 1), north Africa (Morocco; scenario 2) or east (Ethiopia) and north (Morocco) Africa (scenario 3).

To infer the population history of Cameroon goat populations, we analysed HVI *d*-loop region of mtDNA and autosomal SNP markers using Approximate Bayesian Computation (ABC) simulations [54] implemented in DIYABC Ver. 2.1.0 [55]. Both data sets were analysed separately. For mtDNA ABC analysis, a total of 627 HVI region of mtDNA sequences were used and these sequences were grouped based on geographical regions as follows. 1) Cameroon goats; 2) East African goats (Ethiopia and Kenya); 3) North Africa (Morocco, Algeria, Tunisia, Libya and Egypt); 4) Southern Africa (Mozambique, Namibia and Zimbabwe); 5) Middle East and southern Asia (Iran, Iraq, Saudi Arabia and Pakistan). The number of sequences from each country is present at S2 Table. Four demographic scenarios were tested (Fig 1A) assuming that Cameroon goat populations were descended from: east Africa (scenario 1); north Africa (scenario 2); southern Africa (scenario 3); east Africa and north Africa simultaneously (scenario 4). Whereas, for autosomal SNP markers, 1500 SNPs from 848 animals were randomly picked from the 43421 SNPs that qualified the quality control criteria, and used for the ABC evaluation. Three demographic scenarios were tested as indicated in the figure (Fig 1B).

The reference tables were built by 100,000 and one million pods (pseudo-observed data sets) per scenario per set of priors for the autosomal markers and mtDNA, respectively using the default mutation settings. We compared the scenarios by estimating posterior probabilities with the logistic regression method using 0.1% (for mtDNA sequence) and 0.01% (for autosomal SNP markers) of the simulated datasets [56]. For the best model, posterior distributions of the parameters were estimated with a logit-transformed linear regression on the 0.1% (for mtDNA sequence) and 0.01% (for autosomal SNP markers) simulated datasets closest to the observed data [55, 57].

## Results

### The mtDNA d-loop sequence variation and genetic diversity

In all, 93 sequences of the mtDNA *d*-loop region were obtained. These sequences were deposited in GenBank (Accession No. MH621412-MH621504). From 1211 bp of the *d*-loop that was aligned against the caprine reference sequence (Accession No. GU223571; direct submission), 78 variable sites were detected and these defined 83 haplotypes (Table 1). The first haplotype (H1) was shared by the two Cameroon populations analyzed (Djallonke, North-west Highland), whereas H11 was shared by goat populations from Cameroon, Mozambique, Namibia, and Zimbabwe (S3 Table). Similarly, haplotypes H10 and H32 were shared by Cameroon and Ethiopian goats, while haplotype H16 was shared by Cameroon and Kenyan goats. The Cameroon goats did not share any haplotype with goats from west and north Africa, or from outside Africa. The two Cameroon goat populations showed high levels of genetic diversity (average haplotype diversity 0.996 ± 0.006 for Djallonke and 0.993 ± 0.006 for North-west Highland). Average nucleotide diversity was higher for Djallonke goats (*π* = 0.00895 ± 0.00050) than for North-west Highland goats (*π* = 0.00697 ± 0.00058).

**Table 1:** Genetic diversity of indigenous Djallonke and North-west Highland goats in Cameroon, as revealed by mitochondrial DNA (mtDNA) analysis.

Population	N	*S*	H	*H*d ± SD	π± SD	*K*
North-west Highland	48	58	42	0.993±0.006	0.00697±0.00058	8.425
Djallonke	45	63	41	0.996±0.006	0.00895±0.00050	10.807
All sequences	93	78	83	0.995±0.002	0.00805±0.00039	9.730

N = number of samples; S = number of segregating sites; H = number of haplotypes detected; *H*d = haplotype diversity; SD = standard deviation; π = nucleotide diversity; *K* = average number of nucleotide differences.

#### Phylogenetic analysis based on mtDNA

**Fig 2:**
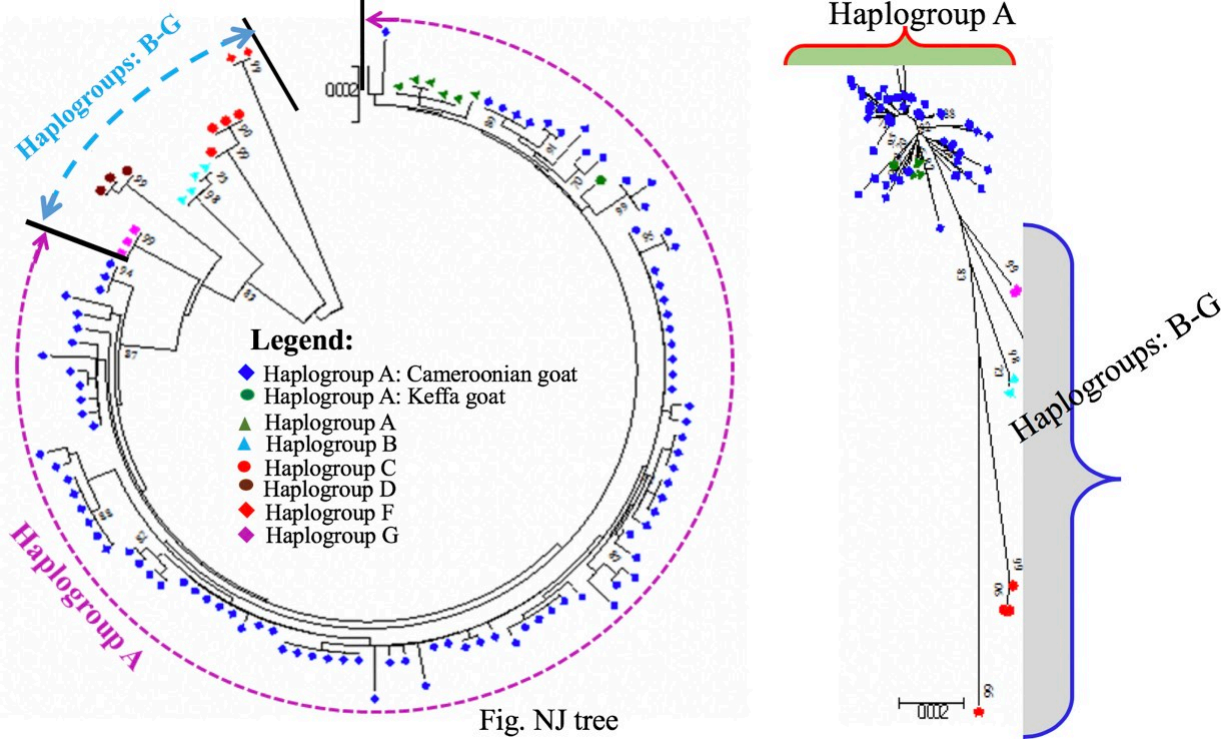
Neighbor-joining (NJ) tree for the Cameroonian goat populations studied.

**Fig 3:**
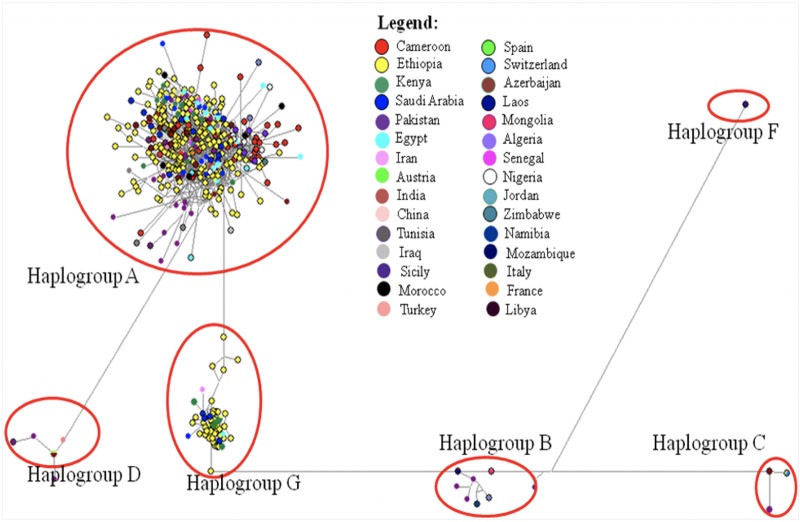
Median-joining (MJ) network graph of Cameroon goat populations and of reference haplotypes from different countries.

The NJ tree constructed for Cameroon goats revealed one well-resolved cluster of haplotypes (Fig 2). The MJ network incorporating 570 reference sequences revealed this cluster to be haplogroup A (Fig 3).

#### Population and historical demographic dynamics

**Fig 4:**
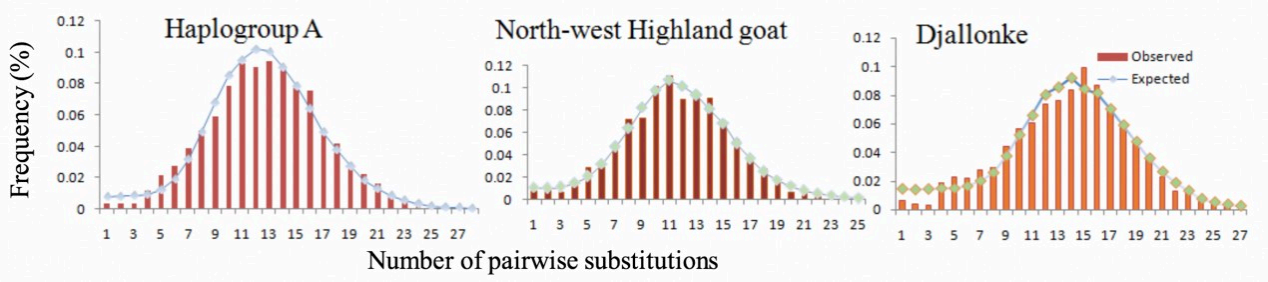
Demographic dynamics revealed by mismatch distribution analysis for haplogroup A goats and for indigenous Djallonke and North-west Highland goat populations in Cameroon.

Mismatch distribution patterns were used to assess the demographic dynamics of the haplogroup revealed by the NJ tree and MJ network (Fig 4). For each population, the mismatch distribution patterns were unimodal and the observed pattern of mismatches did not deviate significantly from that expected in either a spatial or demographic expansion model (Table 2). Similar results, indicating a unimodal pattern of mismatch distribution with the observed pattern that did not deviate significantly from the expected pattern, were observed for haplogroup A, which included all Cameroon goats (Fig 3; Table 2). Negative values were obtained for Fu’s *F*_S_ and Tajima’s *D*, but the latter was not significant (*P* = 0.14200). These results provide evidence of spatial and/or demographic expansion by Cameroon goats.

**Table 2:** Results of neutrality test analysis on indigenous Djallonke and North-west Highland goat populations in Cameroon.

Population	N	S	SSD (*p-*value)	Raggedness index “*r*” (*p*-value)	Tajima’s *D* (*p-*value)	Fu’s *F*_S_ (*p-*value)
North-west Highland	48	58	0.00172 (0.46000)	0.00786 (0.53000)	-1.23868 (0.10000)	-24.82673 (0.00000)
Djallonke	45	63	0.00276 (0.66000)	0.00463 (0.91000)	-0.88535 (0.18400)	-24.56169 (0.00000)
All	93	78	0.00224 (0.56000)	0.00624 (0.72000)	-1.06201 (0.14200)	-24.69421 (0.00000)

N = sample sizes; S = segregating sites; SSD = sum of squared deviations

### SNP genotype analysis

#### Intra-population genetic variability

The proportion of polymorphic SNPs ranged from 95% (North-west Highland) to 99.7% (Barki) (Table 3). The proportion of SNPs deviating significantly from HWE (*P*<0.05) ranged from 1.70% (Iranian goat) to 17.33% (North-west Highland goats). The overall mean value of *H*_O_ was 0.370 ± 0.140 (range 0.335 ± 0.13 (North-west Highland) to 0.401 ± 0.12 (Barki goat)). The overall mean value of *H*_E_ was 0.383 ± 0.127 (range 0.352 ± 0.15 (Central Highland) to 0.422 ± 0.12 (Iranian goats). The average inbreeding coefficient, *F*_IS_, was lowest (-0.02) for Chinese Cashmere goats and highest (0.08) for North-west Highland Cameroon goats. The Cameroon goats had an average *F*_IS_ value of 0.05.

**Table 3:** Single nucleotide polymorphisms and within-population genetic diversity in the study goat populations.

Population	N	*H*_O_	*H*_E_	*F*_IS_	MAF	Mon (%)	Poly (%)	HWE (%) (*P*<0.05)
Djallonke	33	0.348±0.15	0.366±0.14	0.05	0.265	1867(4)	41558(96)	1753 (4.0)
CH	94	0.341±0.15	0.352±0.15	0.03	0.258	1799(4)	41626(96)	2371 (5.5)
NWH	166	0.335±0.13	0.363±0.14	0.08	0.275	2211(5)	41214(95)	7525 (17.3)
Gumez	42	0.376±0.14	0.380±0.13	0.01	0.285	893(2)	42532(98)	1579 (3.6)
Keffa	51	0.353±0.14	0.374±0.13	0.06	0.279	1116(3)	42309(97)	2565 (5.9)
Ambo	119	0.371±0.13	0.379±0.13	0.02	0.289	873(2)	42522(98)	2845 (6.6)
LES	48	0.378±0.14	0.382±0.13	0.01	0.288	668(1)	42757(99)	1581 (3.6)
Afar	49	0.383±0.13	0.391±0.12	0.02	0.299	402(1)	43023(99)	1652 (3.8)
Nubian	47	0.366±0.13	0.395±0.12	0.07	0.303	491(1)	42934(99)	2942 (6.8)
Iranian goat	9	0.392±0.18	0.422±0.12	0.08	0.305	1035(2)	42390(98)	740 (1.7)
Cashmere	108	0.369±0.15	0.363±0.14	-0.02	0.271	1286(3)	42139(97)	1781 (4.0)
Barki	52	0.401±0.12	0.410±0.11	0.02	0.320	129(0.3)	43296(99.7)	1743 (4.0)
Moroc.	30	0.388±0.13	0.411±0.11	0.06	0.317	149(0.3)	43276(99.7)	1591 (3.7)

N = no. of samples, NWCH = North-west Highland; CH = Central Highland; Moroc = Moroccan goat; LES = Long-eared Somali; *H*_*O*_ = observed heterozygosity, *H*_E_ = expected heterozygosity, *F*_*IS*_ = inbreeding coefficient, MAF = minor allele frequency, Mono = monomorphic loci; Ply = polymorphic loci; HWE = deviation from Hardy-Weinberg equilibrium.

**Fig 5:**
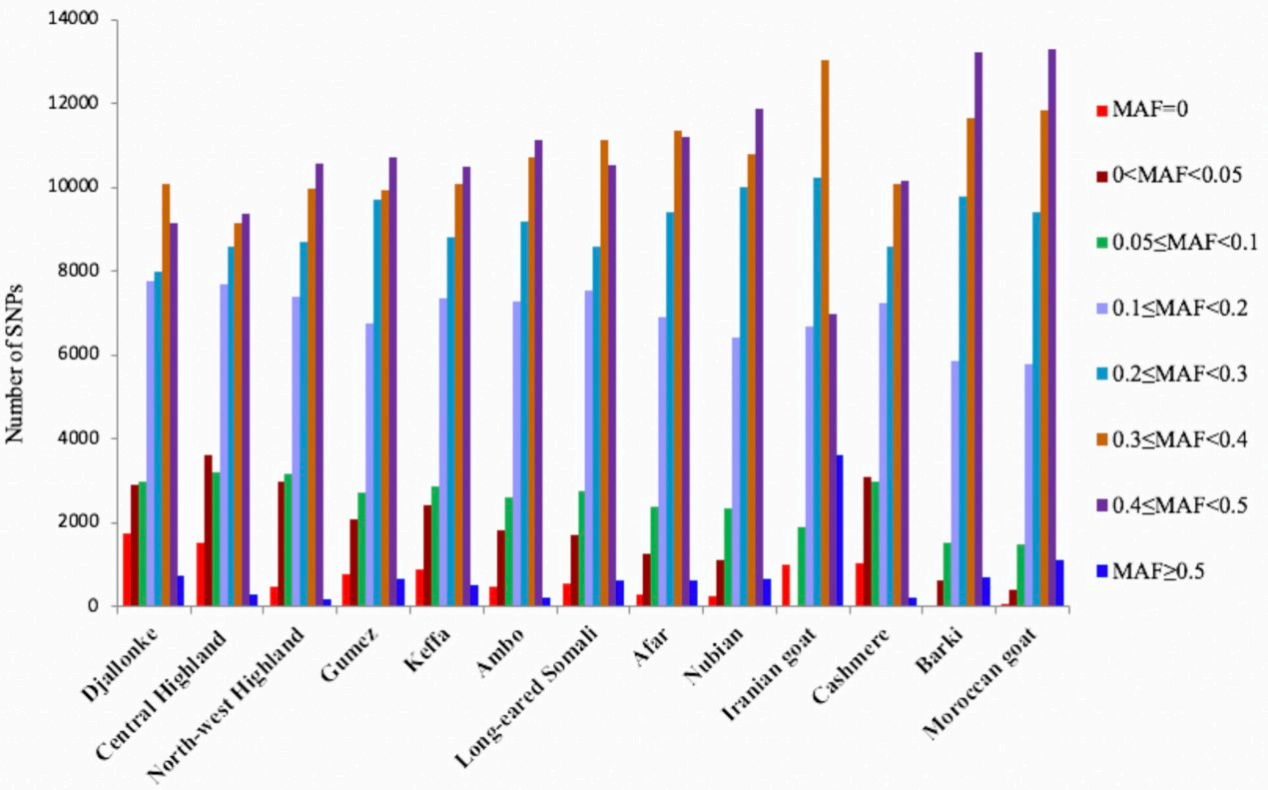
Distribution of minor alleles across 13 goat populations, obtained using 43421 autosomal single nucleotide polymorphism (SNP) markers. MAF = minor allele frequency.

The overall mean MAF was 0.288, and the lowest (0.258) and highest (0.320) values were observed in Central Highland and Barki goats, respectively. Comparatively low estimates of MAF were obtained for Cameroon goats, which was in agreement with the low estimates of heterozygosity. The distribution of MAF showed that percentage of fixed SNPs (MAF = 0.00) ranged from 0.00% in Barki to 4.07% in Djallonke, with an overall mean of 1.62% across populations (Fig 5; S4 Table). Rare alleles (0 < MAF < 0.05) were not observed in Iranian goats. Highly polymorphic SNPs (MAF ≥ 0.30) accounted for 51.14% of the total number of SNPs (range 43.33% for Central Highland to 60.45% for Moroccan goats). The overall mean value of the common alleles (0.10 ≥ MAF ≤ 0.5) was 88.28% (range 80.79% for Central Highland to 95.41% for Moroccan goat), while the overall mean of polymorphic SNPs (MAF > 0.01) was 97.85%.

### Population genetic differentiation and structure

The AMOVA results for the 13 populations grouped by country of origin (Cameroon, Ethiopia, Egypt, Morocco, Iran, and China) revealed that 13.29% (P<0.0001) of the total genetic variation was explained by genetic differences between groups and 81.67% of the variation occurred within individuals (Table 4). Separate analysis of Cameroon goats showed that 1.11% of the variation could be attributed to genetic differences between the three populations included in the analysis (Djallonke, North-west Highland, Central Highland) (S5 Table).

**Table 4:** Analysis of molecular variance (AMOVA) results for 13 goat populations in six population groups (Cameroon, Ethiopian, Cashmere, Moroccan, Egyptian, Iranian).

Source of variation	Degrees of freedom	Sum of squares	Variance component	Percentage of variation
Among groups	5	1617817.146	1256.26969	13.29
Among populations within groups	7	235892.839	197.88555	2.09
Among individuals within populations	835	6912260.984	278.89152	2.95
Within individuals	848	6546877	7720.37382	81.67
Total	1695	15312847.97	9453.42058	

Fixation indices: *F*_IS_ = 0.035; *F*_SC_ = 0.024; *F*_CT_ = 0.133; *F*_IT_ = 0.183; *P*<0.00000 for all indices

Among the Cameroon goats, the overall average *F*_ST_ value was 0.012. This value is lower than the average *F*_ST_ value of Ethiopian goats (0.032) included in this study. The pair-wise *F*_ST_ genetic distance ranged between 0.008 (Djallonke, North-west Highland) and 0.253 (Barki, Central Highland) (S6 Table). Barki goat proved to be the most differentiated from the other study populations (*F*_ST_ = 0.176–0.253). Both *F*_ST_ and Reynolds’ genetic distances showed that Ethiopian goats were more differentiated from Barki goats than from Cashmere and Iranian goats. Cameroon goats were more differentiated from Barki and Cashmere goats than from Iranian goats.

#### Phylogenetic and population structure analyses

**Fig 6:**
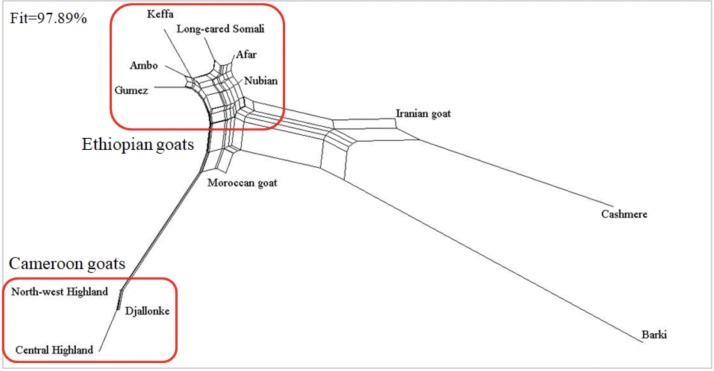
Neighbor-Net network analysis of the 13 goat populations analyzed, based on pair-wise (F_ST_) genetic distances using 43421 autosomal single nucleotide polymorphisms (SNPs).

**Fig 7:**
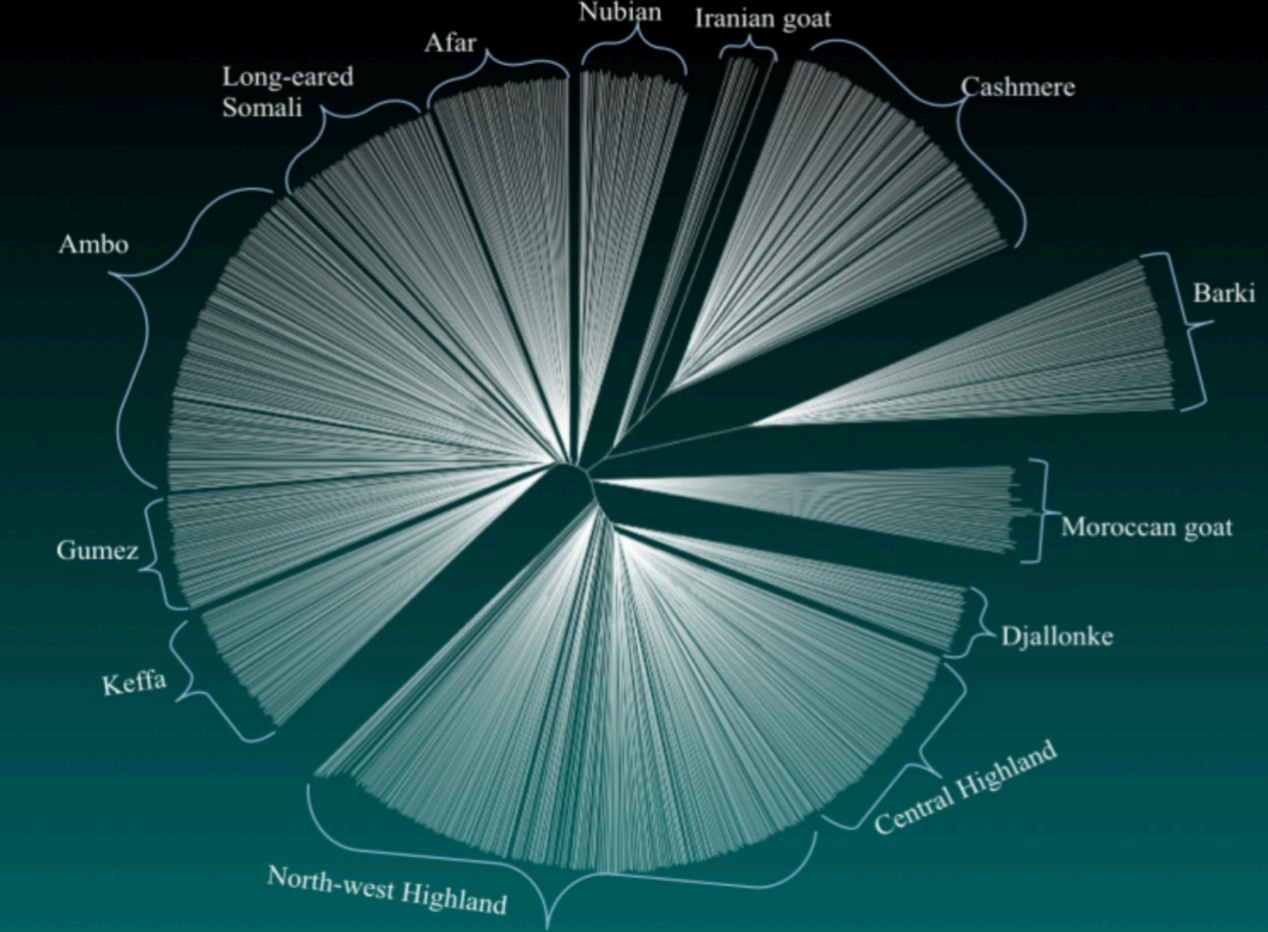
Neighbor-joining (NJ) phylogenetic tree derived from 43421 autosomal single nucleotide polymorphisms (SNPs) of the 13 goat populations analyzed.

The Neighbor-Net network showed that Cameroon goats were highly differentiated from the other goat populations, but were relatively close to Moroccan goats (Fig 6). Compared with Ethiopian goats, very few internal nodes were observed among Cameroon goats and the latter goat populations arose after few population subdivisions. The Neighbor-Net network and NJ phylogenetic trees differentiated the goat populations by country of origin (Cameroon, Ethiopia, Egypt, Morocco, Iran and China). However, there was weak differentiation among the Cameroon goats (Figs 6 and 7). Iranian and Cashmere goats were positioned between Ethiopian and Barki goats. The Neighbor-Net network showed that the three populations (Iranian goat, Cashmere, and Barki) arose from the same clade (Fig 7).

**Fig 8:**
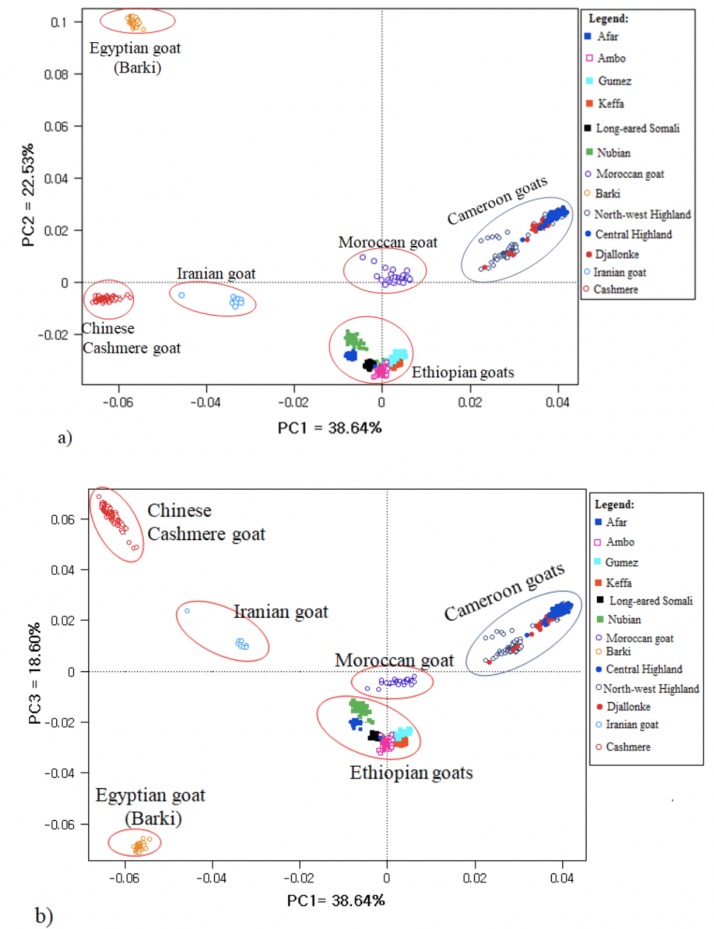
Principal component (PC) analysis plots for the 13 goat populations studied, based on 43421 autosomal single nucleotide polymorphisms (SNPs): a) PC1 and PC-2; b) PC1 and PC3.

In the PCA, principal components PC1 and PC2 accounted for 38.64% and 22.53% of the total variation, respectively (Fig 8A). PCA separated the study populations according to their country of origin, agreeing with the results of the Neighbor-Net network and NJ phylogenetic trees (Figs 6 and 7). In plots of PC1 and PC2, and PC1 and PC3 (Fig 8B), the Moroccan goat was closer to Cameroon goats than to other populations. As also revealed by the NJ tree, Barki and Cashmere goats were distantly clustered from the other populations and the three Cameroon goat populations were poorly differentiated from each other by both PC1 and PC2, and PC1 and PC3.

**Fig 9:**
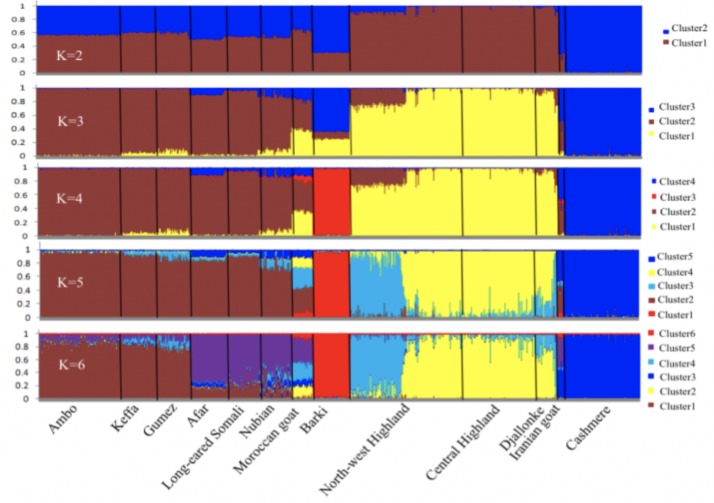
Genetic structure of the 13 study populations evaluated using STRUCTURE.

The results of the STRUCTURE analysis (2 ≤ *K* ≤ 15) are present in Fig 9. In the dataset used, *K* = 6 was the optimal *K* value (S1 Fig) and Cameroon goats demonstrated two major genetic backgrounds. In the first genetic background (at cluster2), Central Highland, North-west Highland, and Djallonke goats had 53.66%, 91.60%, and 63.72% similar genetic background, respectively, in which Moroccan goat shared 16.31% (S7 Table). North-west Highland (43.06%) and Djallonke (34.26%) goats shared the next highest proportion of common genetic background at cluster4, in which Moroccan goat shared 26.26% similar genetic background. Similarly, the goat populations found in hot and arid areas of Ethiopia (Afar, Long-eared Somali, and Nubian) separated from the cohorts found in highland and humid environments (Ambo, Keffa, and Gumez) at *K* = 6. Ethiopian (Afar, Long-eared Somali, and Nubian), Iranian, and Moroccan goats shared 45.43% to 76.16% similar genetic background at cluster5. This is in line with the results of NJ phylogenetic tree and Neighbor-Net network. However, there was only a very small proportion of common genetic background (6.4–10.8%) between Ethiopian and Cameroon goat populations.

**Fig 10:**
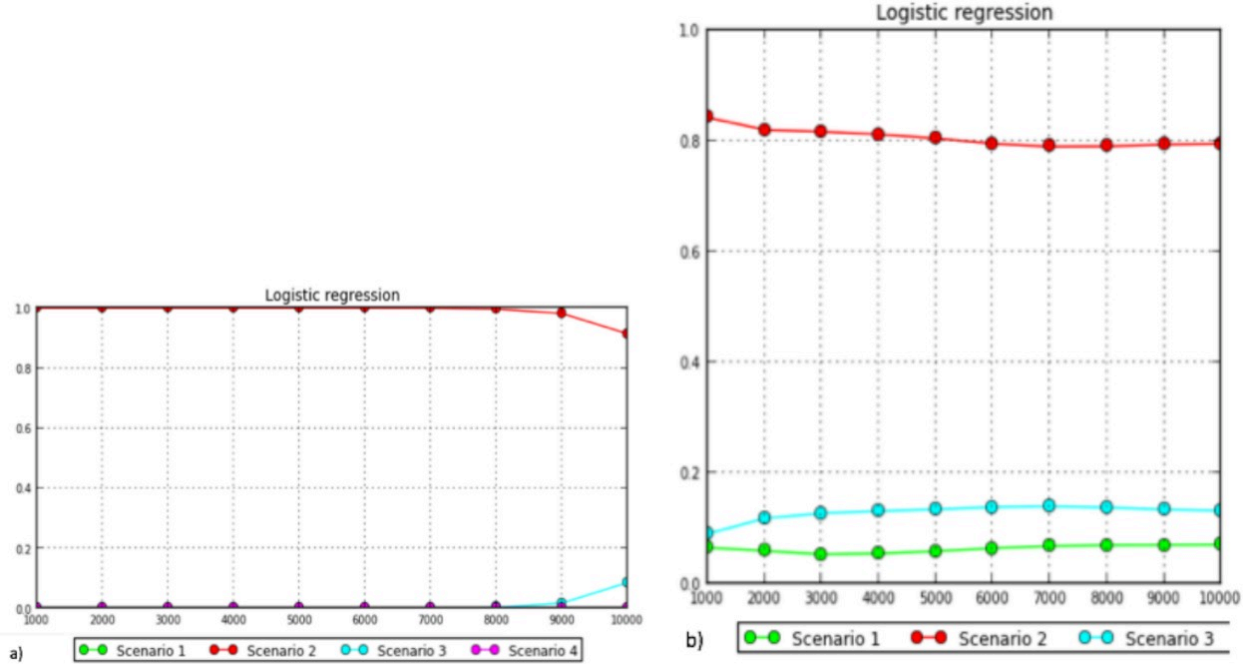
Logistic regression analysis the scenarios inferred using DIYABC: a) mtDNA; b) autosomal SNP markers.

The Bayesian modelling implemented in DIYABC indicated that scenario 2 (Fig 2) was the best fitted model in logistic regression (posterior probability > 0.9126 for mtDNA; > 0.7959 for autosomal SNP markers) (Fig 10; S8 Table) indicating that the southward dispersion of goats from north Africa more explains than the dispersion route from east and southern Africa towards Cameroon. Based on the estimation of posterior distribution for the autosomal SNP markers, the median values of the divergence time showed that the Cameroon goats isolated for the last 506 generations (5% quantile (q050) = 35.9 generations–95% quantile (q950) = 2440 generations) from Moroccan goats (Table 5) which is approximately 1518 years ago assuming three years generation interval per generation.

**Table 5:** Original parameter estimation and statistics (Mean, median, mode and quantiles) of the posterior distribution for the scenarios (in autosomal SNP markers) with the highest posterior probabilities.

Parameter	Mean	Median	Mode	q050	q250	q750	q950
N1	8.01e+03	8.56e+03	9.48e+03	4.32e+03	7.24e+03	9.35e+03	9.85e+03
N2	6.64e+03	7.02e+03	9.05e+03	2.13e+03	5.11e+03	8.59e+03	9.73e+03
N3	2.99e+03	2.16e+03	2.16e+02	1.25e+02	7.32e+02	4.73e+03	8.53e+03
N4	3.02e+02	1.74e+02	5.35e+01	2.51e+01	7.84e+01	3.58e+02	8.53e+02
N5	1.87e+03	1.01e+03	1.35e+02	7.69e+01	3.89e+02	2.56e+03	7.12e+03
N6	1.38e+02	9.79e+01	1.00e+01	1.55e+01	4.71e+01	1.81e+02	3.88e+02
t1	7.94e+02	**5.06e+02**	2.08e+01	3.59e+01	1.90e+02	1.11e+03	2.44e+03
t2	2.26e+03	**1.94e+03**	8.33e+02	3.97e+02	1.07e+03	3.20e+03	5.16e+03
t3	4.89e+03	5.04e+03	5.17e+03	1.88e+03	3.58e+03	6.24e+03	7.64e+03
t4	5.93e+03	6.01e+03	5.81e+03	2.89e+03	4.75e+03	7.23e+03	8.66e+03
t5	6.50e+03	6.54e+03	6.50e+03	3.32e+03	5.29e+03	7.88e+03	9.16e+03
td	8.13e+03	8.55e+03	9.95e+03	4.85e+03	7.18e+03	9.44e+03	9.92e+03
NA	2.95e+03	2.17e+03	2.61e+02	1.51e+02	8.94e+02	4.62e+03	8.24e+03

N1-N2-N3-N4-N5-N6 = Cameroon-Ethiopian-Moroccan-Barki-Iranian-Cashmere goat populations effective population sizes; NA = effective population size of the starting population; t1 = time of divergence of Cameroon goats from Moroccan goats in generations (3 years per generation in goats); td = divergence time from the starting population.

## Discussion

### The *d-loop* sequence analysis

In this study, we analyzed mtDNA *d-*loop sequences of two Cameroon indigenous goat populations (Djallonke and North-west Highland) to assess their maternal origin and historical demography. We observed 83 haplotypes defined by 78 variable sites. In Nigeria, 68 haplotypes generated from 68 polymorphic sites have been reported from 110 sequences in three goat populations (two Nigerian goat populations and one South African Kalahari Red goat population) [58]. In Democratic Republic of Congo (DRC), 56 haplotypes defined from 124 polymorphic sites of 111 animals of three indigenous goat populations have been detected [59]. In east Africa, 29 haplotypes have been identified in 60 sequences of Kenyan indigenous goats and 231 haplotypes have been defined by 174 polymorphic sites from analysis of 309 individuals from 13 Ethiopian goat populations [20, 23]. In north Africa, 40 haplotypes defined from 64 polymorphic sites have been observed in 44 individuals of three Moroccan goat populations [60]. The difference in number of haplotypes detected in these studies could be related to differences in number of populations and sample size employed, variations in the size of the amplified region in mtDNA, and narrowness/wideness of the gene pool in the countries studied. In the present study, we observed 33 median vectors in the MJ network (data not shown). These median vectors could represent unsampled haplotypes or haplotypes that were introduced into the study region but became extinct either upon arrival or afterwards [20]. The relatively low number of median vectors observed could also be because of the presence of a single haplogroup (haplogroup A) arriving in the region.

To evaluate the genetic relationships between the 83 Cameroon goat haplotypes, we constructed an NJ tree and MJ network. The clustering pattern of the haplotypes revealed one well-resolved haplogroup lacking a phylogeographic structure (Figs 2 and 3). Further analysis incorporating reference haplotypes revealed it to be haplogroup A. This haplogroup has previously been reported in Nigeria [22, 58] and Senegal [22, 24]. In contrast, Okpeku et al. [61] suggested the presence of multiple maternal origins of goats in Nigeria following the high degree of genetic admixture observed. However, the results of MJ network and mismatch distribution analyses obtained in that study did not implicitly support the suggested multiple origin, but rather a haplogroup.

To provide insights into the route of dispersal of goats towards Cameroon, we included an additional 570 HVI sequences from 29 countries downloaded from GenBank (S3 Table) and 182 unpublished sequences generated from goats in DRC and Sudan in our analysis. The results showed that Cameroon goats share haplotypes with goats from southern Africa (Mozambique, Namibia, Zimbabwe), east African (Sudan, Ethiopia, Kenya), and DRC. Similarly, the goat populations in Kenya, Ethiopia, Sudan, and DRC share 22 haplotypes. These findings indicate that the initial gene flow or route of dispersion could be from Egypt following the Nile Delta to Sudan and Ethiopia, and then to Cameroon. Based on the archaeological evidence, goats arrived in north Africa from the Fertile Crescent in 5000 BC [18] and are believed to have dispersed southwards following the Nile valley into Sudan and Ethiopia [62]. On the other hand, the southward dispersion of the livestock species from Sahara and Maghreb regions around 2000 BC was restricted by tsetse infestation [63–65]. This dispersion was limited to the Nile Valley route to the south and a tsetse-free corridor to the east along the foothills of the Ethiopian highlands [66, 67]. In studies using modern DNA, the shared haplotypes (all from haplogroup A) detected in Ethiopian and Egyptian goats [20] support the archaeological evidence. The dispersion of goats among Cameroon, east Africa, and southern Africa countries (as supported by the shared haplotypes among goats from Cameroon and eastern and southern Africa countries) could be associated with the movement of the Bantu speaking population. The linguistic and archaeological inferences indicate that the Bantu speaking population started moving to the Great Lake (east) and then to southern Africa [68–70] from eastern Nigeria and west Cameroon [71–74] around 3000–5000 years ago [75–78]. Recent SSR and genome-wide high density SNP chip array-based analyses have also revealed a similar dispersion route of the Bantu speaking population [79, 80]. In goats, it has been postulated that the West African Dwarf goat family could have influenced the genetic composition of east African goats, particularly those found in north-west, west, and south-west parts of Ethiopia [35]. This could possibly be linked to movement of the Bantu speaking population.

In the present study, no shared haplotype was observed between Cameroon and north and west African goats, despite expected gene transfer from north Africa via the Niger valley and Atlantic coastline to Cameroon [19]. This could possibly be due to the few number of reference sequences from north and west Africa used in this study (S2 Table). Results of the population admixture and the approximate Bayesian computation (ABC) better explained the gene flow from north Africa to Cameroon and west Africa in later times (described in later section).

Our mismatch distribution analysis revealed a unimodal pattern for each population and haplogroup A. A similar demographic pattern has been observed in Nigerian goats [58, 61], but not in Ethiopian [20] and Somalian indigenous goats [24], which show a bimodal pattern. This suggests differences in demographic history between goat populations found in Cameroon and west Africa and those from east Africa. It also suggests a single major expansion event of goats into central (Cameroon) and west Africa regions.

### The SNP chip analysis

In the present study, the results for the Forest goat population from Cameroon showed that most (95.41%) of the markers for this population was monomorphic loci. This could be explained by small population size, limited gene flow to the forest environment, and/or the tsetse fly challenge for immigrant animals. The other possible reason could also be ascertainment bias [81], such that the SNP chip array may not be able to appropriately serve for goats found in the forest environment. A similar finding (99.6% monomorphic loci) has been reported for Swiss Ibex [81]. The accumulation of monomorphic loci observed for the Forest goat population in our study has an implication on survival of the population and appeals a need to design and implement conservation and breeding strategies to protect the population from the risk of extinction. However, whole-genome scanning may provide a fuller picture of the status of the population.

#### Intra-population genetic variability

A low overall mean value of fixed SNPs (1.62%) was observed in this study (Fig 5; S4 Table). The overall average value of rare alleles (0.01 ≤ MAF ≤ 0.05) (4.26% of 43421 SNPs) was higher than in Italian goats (0.46% of 51136 SNPs) [82]. Cashmere and Cameroon goats had the highest percentages of rare variants. This could be because of balancing selection [83–84], whereby Cameroon goats could be naturally selected to adapt to the humid environment and Cashmere goat is a specialized breed, artificially selected for successive generations for production of cashmere fiber. Low estimates of rare alleles were recently reported in Sudanese goats (1.8% for Desert goat and 3.1% for Taggar) [31]. In the present study, we observed a relatively low level of genetic diversity in Cameroon goats, while Barki, Moroccan, and Iranian goats had the highest heterozygosity estimates (Table 3). The lack of heterozygosity in the three Cameroon goat populations analyzed could be because of the high level of inbreeding, as also indicated by the high proportion of SNPs deviating from HWE (P≤0.05) (Table 3), or because of sampling bias, small flock size, and poor breeding and flock management strategies.

#### Population genetic differentiation and structure

Based on AMOVA, the genetic variation (13.29%) observed among the goat populations in Cameroon, Ethiopia, Egypt, Morocco, Iran, and China (Table 4) is similar to the variation (11.86%) reported for Angora goats in South Africa, France, and Argentina genotyped with 50K SNP chip array [85]. In a separate analysis of Cameroon goats, we observed 1.11% variation between the populations (S5 Table). This is much lower than the variation reported for Sudanese goats (6.96%) [31] and Italian goats (7.49%) [82]. The low genetic differentiation we observed between Cameroon goat populations could be attributed by intermixing of animals between households and villages and across geographical regions (S6 Table), suggesting to in place animal regulatory strategy. On the other hand, most of the variation is explained by within population variation that could be contributed by uncontrol mating practices by smallholder farmers in Cameroon. Within population selection breeding may help to benefit from the within variation. It can also complement for the community based breeding program (CBBP) in Cameroon. CBBP, in which selection within a population is exercised, provides a good framework for the implementation of genomic selection in small holder systems [86]. In this study, the pairwise *F*_ST_ genetic distance showed that Moroccan goat was closest to Ethiopian goat (S6 Table). This might be because the goats descended from the same origin at the same time, but followed different directions to arrive in the Horn of Africa (Ethiopia) and Morocco. The other possible reason could be connected to the east African Pastoral Neolithic dynamics in which the environmental change and tsetse challenge occurred in the Sahara and north Africa region caused to spread the livestock to east Africa before 2000 BC [87]. However, further investigation is required before firm conclusions can be drawn. Conversely, very high differentiation (*F*_ST_ = 0.176) was observed between Barki and Moroccan goats. This finding is strengthened by the absence of any signature of shared maternal haplotypes between these two populations. In line with this, the MJ network generated with modern maternal DNA showed that the Moroccan lineages were not derived from Egyptian lineages, as would otherwise be expected assuming a terrestrial route for dispersal of the species throughout northern Africa, rather via the Mediterranean routes [88]. Former large-scale mitochondrial analyses [22, 89] have shown that the gene flow towards Morocco did not follow the terrestrial routes. Instead, mtDNA analysis has indicated that the gene flow towards Morocco was via the Mediterranean Sea route, rather than the terrestrial route from Egypt [30].

In the present study, the Neighbor-Net network (Fig 6), the NJ tree (Fig 7) and PCA (Fig 8) differentiated the goat populations based on country of origin. No internal node was detected among Cameroon goats in the Neighbor-Net network, suggesting that the existing populations are intermixed, as supported by the mtDNA and structure results. This indicates that there are no unsampled or lost population(s) from which the existing populations arose [47]. The STRUCTURE analysis also showed that Keffa and Gumez goats (but not other Ethiopian goats) share a considerable proportion of genetic background (10.82% and 6.44%, respectively) with North-west Highland and Djallonke goats of Cameroon at cluster4, *K* = 6 (Fig 9; S7 Table). This observation is in agreement with the shared haplotypes detected (S3 Table). A recent genome-wide analysis of global goats that clustered east and central African goats together [90] strengthens this observation.

Supporting indications provided by the pairwise *F*_ST_ and Reynolds’ genetic distances (S6 Table), the NJ tree showed that Ethiopian goats were closer to Iranian and Cashmere goats than to Barki goats from Egypt (Fig 7). This is in line with our previous finding of shared haplotypes between Ethiopian and Saudi Arabian goats, which led us to suggest that one possible route of introduction of domestic goats to Ethiopia and the Horn of Africa was via Saudi Arabia, crossing the Red Sea [20]. The STRUCTURE output (*K* = 6) showed that Moroccan and Iranian goats shared 37.83% and 45.43% genetic background, respectively, with Ethiopian goats (Afar, Long-eared Somali, and Nubian) found in hot and arid environments (S7 Table). However, the connection between Cashmere and Ethiopian goats still needs further investigation. Among the other Ethiopian goat populations, Nubian and Afar goats shared 7.51% and 6.08% genetic background with Cashmere goats at cluster3, *K* = 6. This observation support the results obtained with NJ phylogenetic tree and Neighbor-Net network (Figs 6 and 7).

Among the goat populations analyzed, Barki goat (Egypt) and Cashmere goat (China) demonstrated the highest proportion of pure genetic background. The latter has undergone long-term artificial selection for production of Cashmere fiber [91] and could therefore be more genetically homogeneous. Similarly, Barki goat could be naturally selected for adaptation to the extreme arid environment in the western desert in Egypt [92]. In fact, selection signatures that harbor genes associated with adaptation to desert stress have been identified in Barki goat [8].

#### Approximate Bayesian Clustering (ABC) simulation

The posterior distribution analysis of ABC in the current study indicated that the second scenario in both data sets (mtDNA and autosomal SNP markers) best explains the goat population dynamics towards Cameroon. This indicates that the second route of dispersion of goats in Cameroon and west Africa could be from north Africa in later times (time of most common ancestor (TMCA) ~1518 YA; *t*_1_; Table 5). Worth noting is that TMCA was calculated considering at least three years generation interval of goat [93–94]. This goes in line with the phylogenetic net-work (Fig 6) and admixture outputs that demonstrated 43% common genetic background of Moroccan goats with Cameroon goat populations at cluster 2 and 4 (Fig 9; S7 Table). A recent genome-wide analysis also showed extensive gene flow (*N*_m_ = 25) amongst the goat populations in Cameroon, Morocco and Ethiopia [90]. In same posterior distribution analysis, the median values of divergence time indicated that Cameroon goats were isolated from their east African cohorts after 1940 generations (5% quantile (q050) = 397 generations–95% quantile (q950) = 5160 generations), which is equivalent to 5820 YA (*t*_2_; Table 5). This period earlier than the period of Bantu speaking people movement which was believed happened between 3000–5000 YA [75–78] but later than the arrival time (5000 BC) of goats in north Africa from center of domestication [18]. However, the gene pool arrived to Cameroon and west Africa region from eastward during earlier times could be influenced by tsetse challenge or could be very small. This is supported by low proportion of genetic background (6.4–10.8%) Ethiopian goats shared with Cameroon goats at cluster4 (Fig 9; S7 Table).

In conclusion, this study provided insights about the population history and structure of Cameroon goats. The maternal DNA information revealed only haplogroup A detected in Cameroon. Population differentiation and admixture analyses showed that Cameroon goats appeared to have very low genetic differentiations and have two major genetic backgrounds, with different proportions, but that all three populations analyzed had been intermixed. The goats in Cameroon could have been dispersed from north and east Africa at different times. The haplotype analysis of mtDNA showed the initial dispersion of goats to Cameroon and central Africa from north-east Africa following the Nile Delta as supported by archaeological, social anthropology and modern human molecular (DNA) evidences. Whereas, the approximate Bayesian computation (ABC) indicated that Cameroon goats were separated from Moroccan goats after 506 generations in later times (~1518 years ago). However, including more mtDNA sequence data from north Africa may warrant to arrive firm conclusion. The differentiation of Barki goat from the other 12 goat populations analyzed shows that Barki goat could be representative of populations found in desert environments for the study of drought tolerance. Interestingly, the autosomal genomic DNA indicated a common origin for Moroccan, Ethiopian and Iranian goats, but this requires further study based on ancient and modern DNA.

## Supporting information

S1 Fig Values of cross validation error plotted against *K* as generated with STRUCTURE. (TIFF)

S1 Table GPS coordinates of Cameroon goat samples included in the study. (XLSX)

S2 Table Reference sequences employed for haplogroup analysis. (DOCX)

S3 Table Haplotype summary of the goat populations of Cameroon and 29 countries: based on the HVI region of the *d*-loop. (XLS)

S4 Table Distribution of minor allele frequency (MAF) generated from 43421 autosomal SNPs. (DOCX)

S5 Table Analysis of molecular variance (AMOVA) for Cameroon goats only using 43421 autosomal SNPs. (DOCX)

S6 Table Pairwise genetic distances (*F*_ST_) (below diagonal) and Reynolds’ distances (above diagonal) among the goat populations studied. (DOCX)

S7 Table Proportion of the different genetic backgrounds observed in the study populations as revealed by Admixture analysis for *K* = 6. (DOCX)

S8 Table Logistic regression analysis of posterior probabilities (confidence intervals) for the scenarios modelled in DIYABC based on: a) mtDNA simulated for 1,000,000 data set; b) Autosomal markers simulated for 100,000 data set.
(DOCX)
