# Supplementary figures and images for: Genome-wide diversity and demographic dynamics of Cameroon goats and their divergence from east African, north African, and Asian conspecifics
Source: PLoS One. 2019 Apr 19;14(4):e0214843. doi: 10.1371/journal.pone.0214843 (PMC6474588; doi:10.1371/journal.pone.0214843)

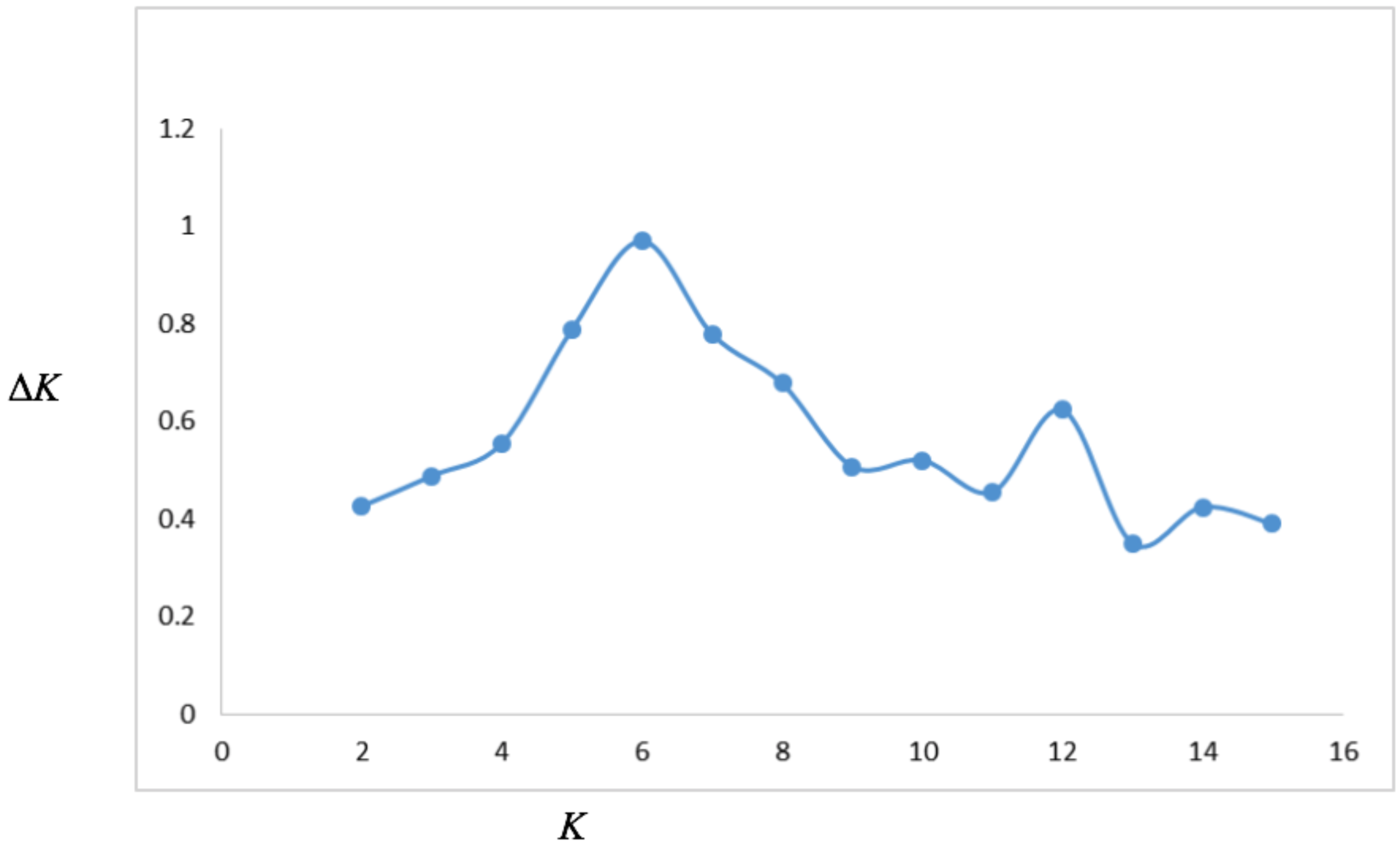

Supplement: S1 Fig — (TIFF) [file pone.0214843.s001.tiff]
