# Supplementary material for: Genome-wide diversity and demographic dynamics of Cameroon goats and their divergence from east African, north African, and Asian conspecifics
Source: PLoS One. 2019 Apr 19;14(4):e0214843. doi: 10.1371/journal.pone.0214843 (PMC6474588; doi:10.1371/journal.pone.0214843)
Supplement: S2 Table — (DOCX) [file pone.0214843.s003.docx]

S2 Table. Reference sequences employed for haplogroup analysis

| **Country of origin** | **Sample size** | **Haplogroup** | **Accession number** | **Author** |
| --- | --- | --- | --- | --- |
| Ethiopia | 306 | A, G | KY747687-KY747993 | [1] |
| Iraq | 7 | A | AJ317762-68 | [2] |
| Iran | 25 | A, G | EF617945, EF617863-EF618084 | [3] |
| Saudi Arabia | 43 |  | AJ317752-59; EF618309-45 | [2-3] |
| Egypt | 26 | A, G | AJ317780-83; AJ317795-801; EF617711-28 | [2-3] |
| Nigeria | 12 | A | AJ317810-811; AJ317823-25; EP618246-52 | [2-3] |
| Turkey | 1 | G | EF618535 | [3] |
| Austria | 1 | D | EF617701 | [3] |
| Mongolia | 1 | B | AJ317833 | [2] |
| Azerbaijan | 1 | B | EF617706 | [3] |
| Jordan | 1 | A | EF618200 | [3] |
| France | 1 | A | EF617779 | [3] |
| Italy | 1 | A | EF618134 | [3] |
| Switzerland | 1 | C | AJ317838 | [3] |
| Spain | 1 | C | EF618413 | [3] |
| Namibia | 4 | A | EF618242- 5 | [3] |
| Zimbabwe | 4 | A | AJ317802-803; EF618545- 6 | [2-3] |
| Mozambique | 8 | A | AJ317804-809; EF618240- 1 | [2-3] |
| Senegal | 3 | A | AJ317816-18 | [2] |
| Tunisia | 6 | A | AJ317789-794 | [2] |
| Libya | 1 | A | EF618220 | [3] |
| Algeria | 3 | A | AJ317777-79 | [2] |
| Morocco | 6 | A | AJ317784 -88; EF618233 | [2-3] |
| Kenya | 58 | A, G | KP120622-KP120681 | [4] |
| China | 3 | B, C, D | DQ121578, DQ188892, DQ188893 | [5] |
| Laos | 1 | B | AB044303 | [6] |
| Pakistan | 40 | A,B,C,D | AB110552–AB110591 | [7] |
| Sicily | 2 | F | DQ241349; DQ241351 | [8] |
| India | 3 | A, D | AY155721, AY155708, AY155952 | [9] |

# References

1. Tarekegn GM, Tesfaye K, Mwai OA, Djikeng A, Dessie T, et al. Mitochondrial DNA variation reveals maternal origins and demographic dynamics of Ethiopian indigenous goats. Ecol Evol 2018; 8(3):1–11. https://doi.org/10.1002/ ece3.3710.

2. Luikart G, Gielly L, Excoffier L, Vigne JD, Bouvet J, Taberlet P. Multiple maternal origins and weak phylogeographic structure in domestic goats. Proceedings of the National Academy of Science USA, 2001; 98: 5927–5932.

# 3. Naderi S, Rezaei HR, Taberlet P, Zundel S, Rafat SA, Naghash HR, et al. Large-scale mitochondrial DNA analysis of the domestic goat reveals six maternal lineages with high haplotype diversity. PLoS ONE 2007; 10:e1012.

4. Kibegwa FM, Githui KE, Jung’a JO, Badamana MS, Nyamu MN. Mitochondrial DNA variation of indigenous goats in Narok and Isiolo counties of Kenya. J. Anim. Breed. Genet 2015; 133(3):238-47.

5.Liu RU, Yang GS, Lei CZ. The Genetic Diversity of mtDNA D-loop and the Origin of Chinese Goats. Acta Genetica Sinica 2006; 33 (5):420–428

6. Mannen H, Nagata Y, Soichi Tsuji S. Mitochondrial DNA reveal that domestic goat (*Capra hircus*) are genetically affected by two subspecies of bezoar (*Capra aegagurus*). Biochemical Genetics 2001; 39 (5/6).

7. Sultana S, Mannen H, Tsuji S. Mitochondrial DNA diversity of Pakistani goats. Anim Genet 2003; 34(6): 417–421.

8. Sardina MT, Ballester M, Marmi J, Finocchiaro R, van Kaam JBCHM, Portolano B and Folch JM, 2006. Phylogenetic analysis of Sicilian goats reveals a new mtDNA lineage. Anim Genet 37(4): 376–378.

9. Joshi MB, Rout PK, Mandal AK, Tyler-Smith C, Singh L, Thangaraj K. Phylogeography and origin of Indian domestic goats. Molecular Biology and Evolution 2004; 21: 454–462.
