# Supplementary material for: Genome-wide diversity and demographic dynamics of Cameroon goats and their divergence from east African, north African, and Asian conspecifics
Source: PLoS One. 2019 Apr 19;14(4):e0214843. doi: 10.1371/journal.pone.0214843 (PMC6474588; doi:10.1371/journal.pone.0214843)
Supplement: S4 Table — (DOCX) [file pone.0214843.s005.docx]

| Population\MAF | MAF=0 | | 0<MAF<0.05 | | 0.05≤MAF<0.1 | | 0.1≤MAF<0.2 | | 0.2≤MAF<0.3 | | 0.3≤MAF<0.4 | | 0.4≤MAF<0.5 | | MAF≥0.5 | | 0.10≥MAF≤0.5 | | MAF≥0.30 | | >0.01 | |
| --- | --- | --- | --- | --- | --- | --- | --- | --- | --- | --- | --- | --- | --- | --- | --- | --- | --- | --- | --- | --- | --- | --- |
|  | N | % | N | % | N | % | N | % | N | % | N | % | N | % | N | % | N | % | N | % | N | % |
| Djallonke | 1769 | 4.07 | 2928 | 6.74 | 2989 | 6.88 | 7762 | 17.87 | 7977 | 18.37 | 10075 | 23.20 | 9165 | 21.11 | 760 | 1.75 | 35739 | 82.30 | 20000 | 46.06 | 41656 | 95.93 |
| Central Highland | 1519 | 3.50 | 3601 | 8.29 | 3220 | 7.42 | 7682 | 17.69 | 8587 | 19.77 | 9161 | 21.10 | 9364 | 21.56 | 291 | 0.67 | 35085 | 80.79 | 18816 | 43.33 | 41276 | 95.05 |
| North-west Highland | 464 | 1.07 | 2981 | 6.86 | 3173 | 7.31 | 7411 | 17.07 | 8701 | 20.04 | 9954 | 22.92 | 10569 | 24.34 | 172 | 0.40 | 36807 | 84.76 | 20695 | 47.66 | 42274 | 97.35 |
| Gumez | 786 | 1.81 | 2096 | 4.83 | 2716 | 6.25 | 6776 | 15.60 | 9725 | 22.39 | 9926 | 22.86 | 10717 | 24.68 | 683 | 1.57 | 37827 | 87.11 | 21326 | 49.11 | 42639 | 98.19 |
| Keffa | 888 | 2.04 | 2415 | 5.56 | 2882 | 6.64 | 7355 | 16.94 | 8799 | 20.26 | 10066 | 23.18 | 10495 | 24.17 | 525 | 1.21 | 37240 | 85.76 | 21086 | 48.56 | 42058 | 96.85 |
| Ambo | 479 | 1.10 | 1815 | 4.18 | 2602 | 5.99 | 7270 | 16.74 | 9198 | 21.18 | 10714 | 24.67 | 11117 | 25.60 | 230 | 0.53 | 38529 | 88.73 | 22061 | 50.80 | 42664 | 98.25 |
| Long-eared Somali | 554 | 1.28 | 1697 | 3.91 | 2767 | 6.37 | 7540 | 17.36 | 8578 | 19.75 | 11111 | 25.59 | 10539 | 24.27 | 639 | 1.47 | 38407 | 88.44 | 22289 | 51.33 | 42871 | 98.72 |
| Afar | 307 | 0.71 | 1255 | 2.89 | 2386 | 5.49 | 6912 | 15.92 | 9402 | 21.65 | 11341 | 26.12 | 11189 | 25.77 | 633 | 1.46 | 39477 | 90.91 | 23163 | 53.34 | 43118 | 99.29 |
| Nubian | 257 | 0.59 | 1113 | 2.56 | 2332 | 5.37 | 6409 | 14.76 | 9991 | 23.01 | 10791 | 24.85 | 11873 | 27.34 | 659 | 1.52 | 39723 | 91.47 | 23323 | 53.71 | 43168 | 99.41 |
| Iranian goat | 1015 | 2.34 | 0 | 0.00 | 1896 | 4.37 | 6674 | 15.37 | 10235 | 23.57 | 13018 | 29.98 | 6969 | 16.05 | 3618 | 8.33 | 40514 | 93.30 | 23605 | 54.36 | 42410 | 97.66 |
| Cashmere | 1028 | 2.37 | 3090 | 7.12 | 2984 | 6.87 | 7225 | 16.64 | 8592 | 19.79 | 10099 | 23.26 | 10171 | 23.42 | 236 | 0.54 | 36323 | 83.65 | 20506 | 47.22 | 41516 | 95.60 |
| Barki | 0 | 0.00 | 645 | 1.49 | 1541 | 3.55 | 5858 | 13.49 | 9786 | 22.54 | 11660 | 26.85 | 13239 | 30.49 | 696 | 1.60 | 41239 | 94.97 | 25595 | 58.94 | 43400 | 99.94 |
| Moroccan goat | 73 | 0.17 | 422 | 0.97 | 1498 | 3.45 | 5778 | 13.31 | 9405 | 21.66 | 11841 | 27.27 | 13285 | 30.59 | 1123 | 2.59 | 41432 | 95.41 | 26249 | 60.45 | 43352 | 99.83 |
| Average |  | 1.62 | 1850.6 | 4.26 | 2537.4 | 5.84 | 6973.2 | 16.06 | 9152 | 21.08 | 10750.5 | 24.76 | 10668.6 | 24.57 | 789.6 | 1.82 | 38334 | 88.28 | 22208.8 | 51.14 | 42492.5 | 97.85 |

S4 Table. Distribution of minor allele frequency (MAF) generated from 43421 autosomal SNPs
