# Supplementary material for: Genome-wide diversity and demographic dynamics of Cameroon goats and their divergence from east African, north African, and Asian conspecifics
Source: PLoS One. 2019 Apr 19;14(4):e0214843. doi: 10.1371/journal.pone.0214843 (PMC6474588; doi:10.1371/journal.pone.0214843)
Supplement: S5 Table — (DOCX) [file pone.0214843.s006.docx]

S5 Table. Analysis of molecular variance (AMOVA) for Cameroon goats only using 43421 autosomal SNPs

| **Source of variation** | **Sum of squares** | **Variance components** | **Percentage of variation** |
| --- | --- | --- | --- |
| Among populations | 30099.664 | 86.40478 | 1.1148 |
| Among individuals within populations | 2365160.881 | 486.43134 | 6.27596 |
| Within individuals | 2097261.5 | 7177.87456 | 92.60924 |
| **Total** | 4492522.045 | 7750.71068 |  |

Fixation Indices: *F*_IS_ = 0.063 (P<0.00000); *F*_ST_ = 0.011 (P<0.00000); *F*_IT_ = 0.074 (P<0.00000)
