# Supplementary material for: Genome-wide diversity and demographic dynamics of Cameroon goats and their divergence from east African, north African, and Asian conspecifics
Source: PLoS One. 2019 Apr 19;14(4):e0214843. doi: 10.1371/journal.pone.0214843 (PMC6474588; doi:10.1371/journal.pone.0214843)
Supplement: S6 Table — (DOCX) [file pone.0214843.s007.docx]

S6 Table. Pairwise genetic distances (*F*_ST_) (below diagonal) and Reynolds’ distances (above diagonal) among the goat populations studied

| Population | Afar | Ambo | Keffa | Gumez | Long-eared Somali | Nubian | Moroccan goat | Barki | North-west Highland | Central Highland | Djallonke | Iranian goat | Cashmere |
| --- | --- | --- | --- | --- | --- | --- | --- | --- | --- | --- | --- | --- | --- |
| Afar | 0 | 0.030 | 0.047 | 0.040 | 0.019 | 0.023 | 0.052 | 0.245 | 0.116 | 0.151 | 0.126 | 0.070 | 0.183 |
| Ambo | 0.029 | 0 | 0.032 | 0.024 | 0.031 | 0.031 | 0.062 | 0.272 | 0.112 | 0.144 | 0.123 | 0.101 | 0.214 |
| Keffa | 0.045 | 0.031 | 0 | 0.037 | 0.042 | 0.043 | 0.070 | 0.282 | 0.116 | 0.152 | 0.129 | 0.120 | 0.238 |
| Gumez | 0.039 | 0.023 | 0.036 | 0 | 0.042 | 0.034 | 0.060 | 0.269 | 0.105 | 0.141 | 0.118 | 0.108 | 0.226 |
| Long-eared Somali | 0.019 | 0.030 | 0.041 | 0.040 | 0 | 0.027 | 0.060 | 0.264 | 0.119 | 0.155 | 0.132 | 0.093 | 0.210 |
| Nubian | 0.022 | 0.030 | 0.042 | 0.033 | 0.026 | 0 | 0.042 | 0.239 | 0.102 | 0.136 | 0.111 | 0.066 | 0.179 |
| Moroccan goat | 0.050 | 0.058 | 0.066 | 0.056 | 0.057 | 0.041 | 0 | 0.213 | 0.062 | 0.090 | 0.070 | 0.072 | 0.193 |
| Barki | 0.197 | 0.214 | 0.220 | 0.212 | 0.209 | 0.193 | 0.176 | 0 | 0.306 | 0.339 | 0.304 | 0.208 | 0.304 |
| North-west Highland | 0.104 | 0.100 | 0.104 | 0.095 | 0.107 | 0.092 | 0.058 | 0.235 | 0 | 0.012 | 0.008 | 0.171 | 0.286 |
| Central Highland | 0.131 | 0.126 | 0.132 | 0.124 | 0.134 | 0.119 | 0.082 | 0.253 | 0.012 | 0 | 0.016 | 0.212 | 0.324 |
| Djallonke | 0.112 | 0.109 | 0.114 | 0.105 | 0.116 | 0.100 | 0.065 | 0.233 | 0.008 | 0.016 | 0 | 0.183 | 0.302 |
| Iranian goat | 0.065 | 0.092 | 0.107 | 0.097 | 0.085 | 0.062 | 0.067 | 0.172 | 0.146 | 0.175 | 0.154 | 0 | 0.092 |
| Cashmere | 0.155 | 0.176 | 0.192 | 0.184 | 0.173 | 0.152 | 0.162 | 0.233 | 0.222 | 0.245 | 0.232 | 0.084 | 0 |
