# Supplementary material for: Genome-wide diversity and demographic dynamics of Cameroon goats and their divergence from east African, north African, and Asian conspecifics
Source: PLoS One. 2019 Apr 19;14(4):e0214843. doi: 10.1371/journal.pone.0214843 (PMC6474588; doi:10.1371/journal.pone.0214843)
Supplement: S7 Table — (DOCX) [file pone.0214843.s008.docx]

S7 Table. Proportion of the different genetic backgrounds observed in the study populations as revealed by Admixture analysis for *K* = 6

| **Population** | **Cluster1** | **Cluster2** | **Cluster3** | **Cluster4** | **Cluster5** | **Cluster6** |
| --- | --- | --- | --- | --- | --- | --- |
| Ambo | 0.8892 | 0.0060 | 0.0049 | 0.0055 | 0.0941 | 0.0002 |
| Keffa | 0.8464 | 0.0083 | 0.0009 | 0.0644 | 0.0796 | 0.0003 |
| Gumez | 0.7802 | 0.0045 | 0.0062 | 0.1082 | 0.1006 | 0.0003 |
| Afar | 0.1891 | 0.0052 | 0.0608 | 0.0080 | 0.7338 | 0.0030 |
| Long-eared Somali | 0.2258 | 0.0035 | 0.0032 | 0.0057 | 0.7616 | 0.0002 |
| Nubian | 0.2073 | 0.0115 | 0.0751 | 0.0855 | 0.6147 | 0.0059 |
| Moroccan | 0.0331 | 0.1631 | 0.0892 | 0.2626 | 0.3783 | 0.0738 |
| Barki | 0.0044 | 0.0043 | 0.0064 | 0.0055 | 0.0035 | 0.9759 |
| North-west Highland | 0.0109 | 0.5366 | 0.0031 | 0.4306 | 0.0173 | 0.0014 |
| Central Highland | 0.0017 | 0.9160 | 0.0006 | 0.0800 | 0.0016 | 0.0001 |
| Djallonke | 0.0090 | 0.6372 | 0.0012 | 0.3426 | 0.0096 | 0.0004 |
| Iranian goat | 0.0036 | 0.0086 | 0.4476 | 0.0273 | 0.4543 | 0.0586 |
| Cashmere | 0.0010 | 0.0009 | 0.9849 | 0.0012 | 0.0110 | 0.0010 |
