# Supplementary material for: Genome-wide diversity and demographic dynamics of Cameroon goats and their divergence from east African, north African, and Asian conspecifics
Source: PLoS One. 2019 Apr 19;14(4):e0214843. doi: 10.1371/journal.pone.0214843 (PMC6474588; doi:10.1371/journal.pone.0214843)
Supplement: S8 Table — Logistic regression analysis of posterior probabilities (confidence intervals) for the scenarios modelled in DIYABC based on: a) mtDNA simulated for 1,000,000 data set; b) Autosomal markers simulated for 100,000 data set. (DOCX) [file pone.0214843.s009.docx]

S8 Table. Logistic regression analysis of posterior probabilities (confidence intervals) for the scenarios modelled in DIYABC based on: a) mtDNA simulated for 1,000,000 data set; b) Autosomal markers simulated for 100,000 data set

| **N** | **Scenario 1** | **Scenario 2** | **Scenario 3** | **Scenario 4** |
| --- | --- | --- | --- | --- |
| 1. **mtDNA** | | | | |
| 1000 | 0.0000 (0.0000,0.0000) | 1.0000 (1.0000,1.0000) | 0.0000 (0.0000,0.0000) | 0.0000 (0.0000,0.0000) |
| 2000 | 0.0000 (0.0000,0.0000) | 1.0000 (1.0000,1.0000) | 0.0000 (0.0000,0.0000) | 0.0000 (0.0000,0.0000) |
| 3000 | 0.0000 (0.0000,0.0000) | 1.0000 (1.0000,1.0000) | 0.0000 (0.0000,0.0000) | 0.0000 (0.0000,0.0000) |
| 4000 | 0.0000 (0.0000,0.0000) | 1.0000 (1.0000,1.0000) | 0.0000 (0.0000,0.0000) | 0.0000 (0.0000,0.0000) |
| 5000 | 0.0000 (0.0000,0.0000) | 1.0000 (1.0000,1.0000) | 0.0000 (0.0000,0.0000) | 0.0000 (0.0000,0.0000) |
| 6000 | 0.0000 (0.0000,0.0000) | 1.0000 (0.9996,1.0000) | 0.0000 (0.0000,0.0004) | 0.0000 (0.0000,0.0000) |
| 7000 | 0.0000 (0.0000,0.0000) | 0.9998 (0.9968,1.0000) | 0.0002 (0.0000,0.0032) | 0.0000 (0.0000,0.0000) |
| 8000 | 0.0000 (0.0000,0.0002) | 0.9970 (0.9354,1.0000) | 0.0030 (0.0000,0.0646) | 0.0000 (0.0000,0.0002) |
| 9000 | 0.0000 (0.0000,0.0060) | 0.9823 (0.6479,1.0000) | 0.0177 (0.0000,0.3521) | 0.0000 (0.0000,0.0060) |
| 10000 | 0.0000 (0.0000,0.1314) | 0.9146 (0.0000,1.0000) | 0.0854 (0.0000,1.0000) | 0.0000 (0.0000,0.1314) |
| 1. **Autosomal markers** | | | | |
| 1000 | 0.0661 (0.0000,0.1357) | 0.8428 (0.7411,0.9446) | 0.0911 (0.0174,0.1647) |  |
| 2000 | 0.0606 (0.0174,0.1037) | 0.8206 (0.7446,0.8966) | 0.1189 (0.0565,0.1813) |  |
| 3000 | 0.0543 (0.0230,0.0856) | 0.8175 (0.7552,0.8798) | 0.1282 (0.0747,0.1818) |  |
| 4000 | 0.0558 (0.0284,0.0832) | 0.8124 (0.7576,0.8671) | 0.1319 (0.0847,0.1790) |  |
| 5000 | 0.0599 (0.0340,0.0858) | 0.8051 (0.7549,0.8553) | 0.1350 (0.0922,0.1778) |  |
| 6000 | 0.0651 (0.0398,0.0904) | 0.7959 (0.7487,0.8431) | 0.1390 (0.0991,0.1789) |  |
| 7000 | 0.0688 (0.0443,0.0932) | 0.7908 (0.7464,0.8352) | 0.1404 (0.1033,0.1776) |  |
| 8000 | 0.0704 (0.0472,0.0937) | 0.7913 (0.7499,0.8327) | 0.1383 (0.1040,0.1725) |  |
| 9000 | 0.0708 (0.0489,0.0928) | 0.7944 (0.7558,0.8330) | 0.1347 (0.1032,0.1663) |  |
| 10000 | 0.0712 (0.0504,0.0921) | 0.7959 (0.7596,0.8323) | 0.1328 (0.1033,0.1624) |  |

N = number of selected data sets closest to the observed data
